# Assessment of the Efficacy of L-Lysine Sulfate vis-à-vis L-Lysine Hydrochloride as Sources of Supplemental Lysine in Broiler Chickens

**DOI:** 10.4061/2010/964076

**Published:** 2010-07-28

**Authors:** Vijay Bahadur, Sudipto Haldar, Tapan Kumar Ghosh

**Affiliations:** Department of Animal Nutrition, Faculty of Veterinary & Animal Sciences, West Bengal University of Animal & Fishery Sciences, 37 Kshudiram Bose Sarani, Kolkata 700037, India

## Abstract

In this study the effects of L-lysine hydrochloride (containing 78.8% available lysine as crystalline lysine) and L-lysine sulfate (containing 51% available lysine in bacterial cell mass) as source of supplemental lysine in broiler chickens was assessed. The basal diet was supplemented with either L-lysine hydrochloride or L-lysine sulfate to meet lysine requirement. Lysine supplementation irrespective of source improved (*P* < .05) live weight and food conversion. Live weight and food conversion ratio of the L-lysine sulfate group was superior (*P* < .05) to the L-lysine hydrochloride group. Supplementation of lysine to the basal diet improved breast meat yield (*P* < .05). Meat protein content and protein accretion increased (*P* < .01) when L-lysine sulfate was supplemented. Nutrient metabolizability, N retention, protein utilization efficiency and live weight gain : lysine intake ratio also improved (*P* < .01) with L-lysine sulfate. A fasting trial conducted after the completion of the feeding trial indicated that the birds receiving L-lysine sulfate retained more of their live weight than the control and the L-lysine hydrochloride dietary groups (*P* < .05). It was concluded that due to the retained bacterial cell mass, L-lysine sulfate may be a superior source of supplemental lysine than L-lysine hydrochloride for broiler chickens.

## 1. Introduction

In standard formulations of broiler diets, lysine is frequently a limiting amino acid and its dietary supplementation is a common practice keeping in view the negative effects of lysine deficiency on growth and feed intake [1]. The conventional form in which lysine is supplemented in broiler diet is lysine hydrochloride (L-lysine.HCl) which contains 78% available lysine. L-lysine sulfate is an alternative to L-lysine.HCl and contains 52% available lysine [1]. Unlike crystalline L-lysine.HCl, L-lysine sulfate contains by-products of carbohydrate fermentation and dried microbial cells [2, 3]. The presence of dried microbial cells imparts additional nutritive value to L-lysine sulfate in the form of sulfur, amino acids other than lysine and phosphorus and in pigs L-lysine sulfate reportedly supplies 18.7 MJ ME/kg and 12% nitrogen [4]. It may, therefore, be hypothesized that these additional nutrients may elicit value addition effects which may be reflected in production and metabolic traits of broiler chickens.

The objectives of the present study were (i) to compare the effects of lysine supplementation in diet either in the form of L-lysine sulfate or L-lysine.HCl on performance and carcass traits of broiler chickens, and (ii) to ascertain the effects of the aforementioned sources of supplemental lysine on intake and metabolizability of nutrients and endogenous losses of nitrogen in broiler chickens due to supplementation of L-lysine.HCl, and L-lysine sulfate. 

## 2. Materials and Methods

### 2.1. Birds and Experimental Design

The experiment was conducted according to the institutional ethical norms. An as-hatched flock of 250 Cobb-400 broiler chickens were used in this study which lasted 40 days. The birds were weighed and placed in a battery type Californian brooder. Ground corn was offered within 12 hours of hatching and on day 1 they were distributed randomly according to body weight into three dietary treatments each consisting of 8 replicates with 10 birds in each replicate. The replicates were placed in cages (48 cm × 60 cm × 48 cm). The starter (day 1–28) and the finisher (day 29–40) diets (Table 1) were offered *ad libitum*. All diets were in mash form. Standard management and vaccination practices were followed.

The dietary treatments consisted of feeding the birds with a basal diet devoid of supplemental lysine (negative control), the basal diet supplemented with L-lysine.HCl and the same supplemented with L-lysine sulfate (manufactured by CheilJedang Corporation, Seoul, Republic of South Korea). 

### 2.2. Live Performance and Carcass Traits

Pen means for food intake, live weight change, and food conversion ratio (FCR, food intake g: live weight gain g) were measured at every 10 days interval. Mortality, if any was recorded, the data was adjusted accordingly. Prior to the start of experimental feeding, 10 birds were killed, and the carcasses were frozen at −20°C. At the end of the experimental feeding (day 40) 10 birds were selected randomly from each of the treatment groups. The selected birds had body weight close to the average body weight of the particular dietary group. Slaughter was performed by mechanical stunning followed by exsanguinations, and the hot carcass weight (with the head, blood, neck, and hocks removed), eviscerated carcass weight, carcass yield (weight of defeathered eviscerated carcass relative to live weight), and yields of breast, frame, legs, and wings were determined. The yields of the latter components were expressed relative to the dressed carcass weight. Breast weight included the breast fillet and the tenders (pectoralis major and minor) and the frame was defined as the eviscerated body frame with the head, neck, wings, legs, and breast removed. The giblet included the liver, heart, lungs and gizzard. Carcasses were stored at −20°C till analyzed for moisture, ash, nitrogen (CP), and crude fat [5]. Protein accretion in carcass in 40 days was also determined. 

### 2.3. Collection and Analysis of Blood

Blood (approximately 10 mL) was collected from the slaughtered birds on day 0 (prefeeding samples) and 10 birds from each dietary treatments on day 40 (post-feeding samples) from the severed jugular vein and the wing vein, respectively. Immediately after collection the tubes containing the blood were placed in ice for 60 minutes for clotting, and the serum was separated from the cells by centrifugation at 2500 × g for 10 minutes (Remi Research Centrifuge, Model R-8C, Remi Research Laboratories, Mumbai, India). The serum was harvested and stored at −20°C in polystyrene tubes till analyzed for glucose, cholesterol, triglyceride, total protein, albumin, urea-N, uric acid, creatinine, and chloride in an automated blood analyzer (Microlab 200, Vital Scientific, Dieren, The Netherlands). Commercial kits (manufactured by Med Source Ozone Bio medicals, Faridabad, India) were employed for these analyses. 

### 2.4. Metabolism Trial and Estimation of Endogenous N Loss

A metabolism trial was conducted between days 32 to 38 taking 10 birds from each dietary treatment. The birds were kept in individual cages, and the amount of food offered and that of the residues left in each cage were measured. The excreta were removed every 2-hour interval and put in self-zipped polyethylene sachets, and the total amount of excreta obtained in a 24 hours period was weighed. The excreta were manually mixed, a subsample measuring 1/20th of the total excreta volume was kept daily in a hot air oven at 80°C for 16 hours, and the dry matter (DM) and organic matter (OM) contents were determined [5]. Another sub sample measuring 1/20th of the total excreta volume was kept everyday in plastic bottles containing dilute (250 mL/L) sulfuric acid. The samples were pooled at the end of the metabolism trial and analyzed for N and CP [5]. The diets were also analyzed similarly for the above nutrients. Nutrient metabolizability (expressed as a coefficient) and retention coefficient of N were calculated. Intake of total phosphorus, lysine, methionine, cysteine, arginine, threonine, and tryptophan was calculated as functions of food intake. Utilization efficiency of dietary protein was determined by calculating the ratio between protein accretion and protein intake and that between the intake of CP and live weight gain. 

Fasting and endogenous N loss were determined at the end of the metabolism trial. The birds were fasted for 48 hours and weighed. The feces voided was collected and analyzed for N. The birds were then refed individually for the next 6 days with tapioca starch which was mixed with the additives and premixes as it was done with the basal finisher diet excepting the amino acids. The period was designated as the “protein withdrawal period.” Live weight change and food intake were recorded daily, and the change in live weight and the mean food intake was calculated at the end. Individual excreta were collected daily, weighed, and frozen till analyzed for N and CP. Blood samples (5 mL) were collected from individual birds at the end of the protein withdrawal period, and the serum was analyzed for total protein and creatinine. 

### 2.5. Statistical Analysis

A completely randomized block design was used to analyze the data. The replicates served as the experimental units, and the data were adjusted according to the number of birds present in each replicate. However, for certain parameters like carcass traits, nutrient metabolism, and endogenous N loss, individual observations were used in calculations. The results were expressed as mean and pooled standard error of mean (SE). Differences in response criteria were determined using the Tukey's test, and all comparisons were made at a probability level of .05. A probability of *P* < .1 was described as trend. 

## 3. Results

### 3.1. Chemical Composition of Experimental Diets

The dietary composition (Table 1) indicated that the basal diet contained adequate CP and ME and except total lysine, concentrations of other amino acids, calcium and available phosphorus were optimum for growth of Cobb 400 birds. The basal starter and the finisher diets of the negative control group were deficient in total lysine by 15 and 11%, respectively, relative to its requirement during the corresponding growth stages. Compared to the basal starter diet, which contained (per kg) 222.3 g CP, 12.77 MJ ME, and 11.5 g total lysine, the starter diet supplemented with L-lysine.HCl contained 224.3 g CP, 12.77 MJ ME, and 13.4 g total lysine per kg. The L-lysine sulfate containing starter diet, on the other hand contained 224.3, 12.83, and 13.36 g total lysine per kg. The basal finisher diet contained 191.6 g CP, 13.36 MJ ME, and 9.5 g total lysine per kg. The corresponding values in the L-lysine.HCl diet were 192.8 g, 13.36 MJ, and 10.67 g per kg, respectively. The L-lysine sulfate finisher diet contained 192.9 g CP, 13.4 MJ ME, and 10.74 g total lysine per kg. Compared to the basal and the L-lysine.HCl supplemented diets, concentration of total phosphorus, methionine, cysteine, arginine, threonine, and tryptophan was marginally higher when L-lysine sulfate was supplemented to the diets. 

Chemical analysis of L-lysine sulfate (Table 2) indicates that it contained comparatively higher quantum of ME than L-lysine.HCl for poultry. Additionally, L-lysine sulfate contained 0.16% phosphorus and amino acids like methionine, cysteine, threonine, tryptophan, arginine, and isoleucine which were absent in L-lysine.HCl. Thus, L-lysine sulfate appeared to have acted as a source of other nutrients apart from lysine *per se*.

### 3.2. Performance and Carcass Traits

Irrespective of source supplementation of lysine improved live weight (*P* = .006), total live weight gain (*P* = .005), and cumulative FCR (*P* = .028) relative to the lysine deficient control group. Performance of the birds fed L-lysine sulfate was superior (*P* < .05) to the L-lysine.HCl dietary group. The cumulative food intake in 40 days was lower (*P* = .028) in the L-lysine sulfate dietary group compared to that in the control and the L-lysine.HCl groups of birds (Table 3).

Weight of the hot carcass and eviscerated carcass as well as carcass yield was not affected by dietary treatments (*P* > .1) although both the above stated parameters were approximately 3% higher in the lysine supplemented birds. Relative to the control group, yield of the frame tended to be higher (*P* = .064) and that of the breast was significantly higher (*P* = .009) in the lysine supplemented groups. Yields of legs, wings and giblets were similar across the dietary treatments (*P* > .1). Relative to the control group of birds supplementation of lysine, irrespective of source, increased yield of frame (*P* = .015) and breast (*P* = .05) calculated as a percentage of live weight. Weight of the wings as a percentage of live weight was, however, lower (*P* = .028) in the L-lysine sulfate group of birds compared to the control and the L-lysine.HCl dietary groups (Table 3).

Dietary treatments had subtle effects on moisture content of meat (*P* > .1). However, meat ash content was higher in the birds receiving L-lysine sulfate (*P* = .038) compared to the other treatment groups. Ether extract content of meat was significantly lower in the birds supplemented with L-lysine.HCl compared to those in the control and the L-lysine sulfate dietary groups of birds (*P* = .024). Protein content of meat increased significantly due to L-lysine sulfate supplementation compared to the control and the L-lysine.HCl groups of birds (*P* = .005). Protein accretion in carcass of the experimental birds between days 0 and 40 was higher (*P* = .0001) in the L-lysine sulfate supplemented group vis-à-vis that in the control and the L-lysine.HCl groups (Table 3). 

### 3.3. Concentration of Serum Metabolites

There were subtle effects (*P* > .1) of dietary treatments on serum concentrations of total protein, albumin, and globulin (Table 4). However, total protein and albumin in serum decreased and that of globulin was higher on day 40 relative to the initial measurement across the dietary treatments (day effect *P* < .05, diet—day interaction effect *P* > .1). Serum concentration of the protein metabolites like creatinine and urea—N were also not affected due to supplementation of L-lysine.HCl and L-lysine sulfate. Uric acid in serum was higher (*P* = .036) in the lysine supplemented birds on day 40 (day—diet interaction *P* < .05). Glucose, cholesterol, and chloride in serum were similar across the dietary treatments (*P* > .1, diet—day interaction effect *P* > .1). 

### 3.4. Nutrient Metabolizability and Protein Utilization Efficiency

Dietary supplementation of lysine as L-lysine sulfate improved (*P* < .01) metabolizability of DM, OM, and CP relative to the control and the L-lysine.HCl supplemented dietary groups (Table 5). Intriguingly, CP and ME intake was similar across the dietary treatments (*P* > .1) although average daily food intake was lower (*P* = .028) in the L-lysine sulfate group than the control and the L-lysine.HCl dietary groups. Intake of total P and other amino acids particularly lysine, methionine, arginine and threonine was significantly higher (*P* = .0001) in the L-lysine sulfate supplemented group. Retention of N in the same dietary group was also higher (*P* = .003) than that in the control and the L-lysine.HCl groups. Protein utilization efficiency, calculated as a ratio between protein accretion and protein intake, improved significantly when L-lysine sulfate was supplemented as the source of supplemental lysine (*P* = .001). Utilization efficiency of dietary protein, energy, and lysine for live weight gain was also better (*P* < .01) in the L-lysine sulfate dietary group compared to that measured in the control and the L-lysine.HCl supplemented group of birds (Table 5).

### 3.5. Fasting and Endogenous N Loss

**Figure 1:**
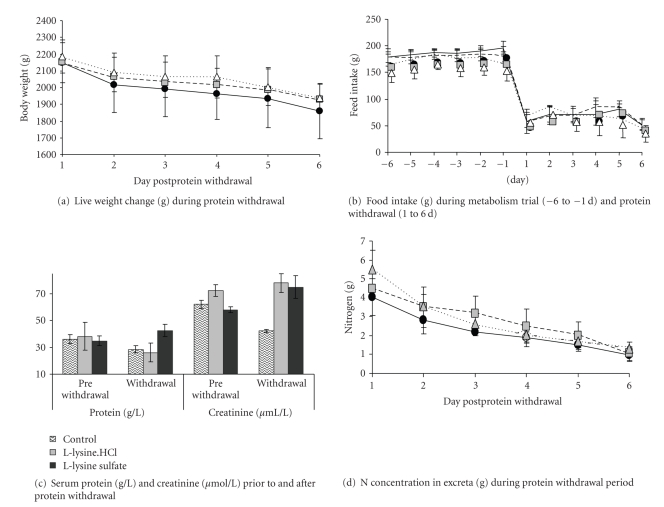
Live weight change, food intake, serum concentrations of total protein and creatinine, and excreta N in experimental broilers. Based on data obtained during the fasting trial conducted at tandem with the metabolism trial (*n* = 10) selected at random from each treatment group. In the line diagrams solid, dashed, and dotted lines, respectively, indicate the control, L-lysine.HCl, and L-lysine sulfate dietary groups.

Live weight of the birds at the start of the fasting was similar across the dietary treatments although at the end of the fasting live weight of the L-lysine sulfate supplemented birds was found to be higher (*P* = .017) than that in the control and the L-lysine.HCl dietary groups (Table 6). Live weight declined identically (*P* > .1) in all dietary groups when protein was withdrawn from diet (Figure 1(a)). Food intake (Figure 1(b)) across the dietary treatments was similar during the period of protein withdrawal (*P* > .1). However, compared to the experimental period, feed intake during the protein withdrawal period was lower (*P* = .0001, diet—day interaction *P* = .078) in all the treatment groups. Serum protein and creatinine (Figure 1(c)) were similar across the dietary treatments at the start of the protein withdrawal (*P* > .1). At the end of the protein withdrawal period serum protein was higher in the L-lysine sulfate fed group compared to the control and the L-lysine.HCl dietary groups (*P* = .028). Serum creatinine, on the other hand, was elevated when lysine was supplemented in diet at the end of the protein withdrawal period (*P* = .0001). Excretion of N through excreta decreased as the protein withdrawal progressed (day effect *P* = .0001). N content in the excreta (Figure 1(d)) was higher in the L-lysine.HCl and L-lysine sulfate dietary groups compared to the control group of birds (diet effect *P* = .002) throughout the protein withdrawal period (day—diet *P* = .028). 

## 4. Discussion

The markedly poorer growth in the control group reinstated the sensitivity of broilers to dietary lysine concentration. Contrary to some earlier investigations [4, 6, 7] the L-lysine sulfate supplemented dietary group performed better than the L-lysine.HCl supplemented group. L-lysine sulfate contained comparatively higher metabolizable energy than L-lysine.HCl due to the presence of dried microbial cells. Moreover, lysine sulfate supplied added quantity of P, and amino acids like lysine, methionine, arginine, and threonine. It is possible that these additional nutrients imparted some edging effect over L-lysine.HCl and enhanced the performance of the birds. Perusal of Table 5 clearly indicated that apart from lysine intake of other amino acids particularly that of methionine was more than 70% higher in the L-lysine sulfate dietary group relative to that in the L-lysine.HCl group of birds. These additional amino acids probably acted synergistically with the lysine to improve performance of the L-lysine sulfate dietary group. 

Dietary lysine concentration affects food intake in birds [8], and chicks attempt to compensate for dietary lysine deficiency by increasing food intake [8]. Daily food intake in the lysine sulfate supplemented dietary group was 3.73% and 2.69% lower than that in the lysine deficient control and L-lysine.HCl supplemented groups of birds. Contrarily, intake of total lysine in the L-lysine sulfate dietary group was approximately 45% and 29% higher compared to that in the control and the L-lysine.HCl dietary groups, respectively. In the L-lysine.HCl group intake of lysine was only 13% higher than that in the lysine deficient control group. Plausibly, supplementation of lysine as lysine sulfate helped the birds to meet their requirement of lysine and other amino acids even at a lower plane of food intake.

The large variation in total lysine intake between the L-lysine.HCl and L-lysine sulfate group despite both the diets being iso-lysine content may be explained by the difference in amino acid composition of both the lysine sources. L-lysine sulfate contained 0.35% additional lysine in the retained bacterial mass which was not there in L-lysine.HCl. It is obvious that the higher intake of the amino acids including that of lysine in the L-lysine sulfate dietary group was due to the presence of the additional amino acids retained in the bacterial cell mass. The present findings were in agreement with an earlier report [9] which indicated that dried microbial cells had amino acid composition comparable to fish meal and its digestibility as a whole and that of its amino acids in particular was rather better than that of fish meal in case of pigs.

Lysine is particularly important in assuring yield of skinless and boneless breast meat [10–12]. Yields of whole carcass and different carcass components were not affected by the source of supplemental lysine although the birds in the L-lysine sulfate group were on a comparatively better plane of nutrition in terms amino acid intake. Nevertheless, despite absence of statistical significance, it is worth mentioning that lysine supplementation, irrespective of source, improved the hot carcass weight and eviscerated carcass weight by 3% over the control group. Overall, the findings indicated that irrespective of the source, when lysine was supplemented to diet, the birds devoted more body weight to skeletal muscle and deposited more protein over there as well as on to the viscera, and L-lysine sulfate seemed to have greater efficacy than L-lysine HCl in this regard. As a result, the birds in the L-lysine HCl and L-lysine sulfate dietary groups, despite the overall carcass yield being similar, exhibited greater yields of breast and frame relative to the control group of birds.

The relationship between abdominal fat pad content and total carcass fat content with dietary lysine is rather ambiguous. The body composition of the experimental broilers indicated that a major shift in metabolism occurred due to lysine supplementation, and the source of supplemental lysine had a significant relationship with the carcass composition of the experimental chickens. The higher carcass fat content in the control group of bird vis-à-vis that in the L-lysine HCl dietary group was indicative of an increased fat synthesis due to a deficient lysine intake as it was reported earlier is [1, 13]. However, the higher body fat content in the L-lysine sulfate dietary group could not be explained. Carcass protein content and protein accretion values revealed that L-lysine sulfate caused greater protein utilization efficiency compared to L-lysine.HCl which may be due to some enhanced metabolic consequences related to intake of lysine [1, 14, 15]. 

Serum biochemical traits often correlate well with the nutritional status of animals [16]. However, in this study serum metabolites did not show any significant correlation with the nutritional status and performance of the birds. The results rather suggested that despite increase in lysine intake serum biochemical indices varied little. Moreover, substituting L-lysine HCl with L-lysine sulfate may not affect the homeostasis of the blood metabolites. 

The most common method for evaluation of food protein and to express N requirements in poultry is the method of ileal digestibility because the ileum is the last segment of the digestive tract where the amino acids are absorbed and used for protein synthesis [17]. Estimation of endogenous N loss is important since the N from endogenous origin leads to underestimation of the amount of the dietary N absorbed and utilized. Earlier works in this regard indicated that estimation of endogenous protein excretion can be measured more accurately when diets containing small quantity of protein was fed to birds instead of a totally protein-free diet [18, 19]. In the present investigation a tapioca starch-based diet containing 2.3 g N/kg DM was used for determination of endogenous N loss. The dietary N concentration was assumed to be conducive to proper estimation of endogenous N loss in the experimental birds. The initial and the final live weight of the birds during this experiment was a reflection of their final body weight on day 40 of the experimental feeding period. The results indicated that the birds supplemented with L-lysine sulfate were at a better plane of nutrition and perhaps had better nutrient reserves which resulted significantly less body weight loss in the said dietary group vis-à-vis the control and the L-lysine.HCl groups during the fasting period. Feed intake did not reach the normal level when feeding resumed after 48 hours. This obviously led to a state of dietary energy deficiency which could not be compensated otherwise. Nevertheless, it was perhaps the additional nutrients consumed by them in the L-lysine sulfate dietary group which helped these birds to preserve more of their body weight during the periods of fasting and tapioca starch feeding. The phenomenon may be described as “catch up” protein growth [20], and this hypothesis is corroborated from the increased protein utilization efficiency in the L-lysine sulfate group during the feeding trial. The endogenous N loss also indicated that the birds supplemented with L-lysine sulfate were more efficient in reducing the fecal N flux and thereby retained a greater proportion of the dietary protein consumed. Plausibly, this was due to a deceleration in protein catabolism or an increase in protein anabolism which augmented protein utilization efficiency in the L-lysine sulfate dietary group. It is hypothesized [9] that some part of the nucleic acid fraction of the dietary bacterial cell mass may be available to animals particularly during the periods of protein deprivation. The present findings also corroborated this hypothesis. The lower serum creatinine concentration in the control group of birds was probably due to decreased urea synthesis occurred as a carry-over effect of lysine deprivation during the experimental feeding. 

The present investigation revealed that L-lysine sulfate may be a better option as a source of supplemental lysine for broilers. Dietary supplementation of L-lysine sulfate may result in higher intake of available phosphorus, total lysine, and other amino acids and may enhance nutrient metabolizability. Increased utilization efficiency of dietary protein and higher body protein accretion were observed when L-lysine sulfate was supplemented in diet and as a consequence live weight gain and feed conversion efficiency may also be improved with L-lysine sulfate. The study finally concluded that the retained bacterial cell mass may exert a value addition effect to L-lysine sulfate and makes it a superior source of supplemental lysine for broiler chickens. 

## Figures and Tables

**Table 1:** Ingredients and chemical composition (dry matter basis) of the basal diet.

	Starter diet (1–24 days)	Finisher diet (25–40 days)
Ingredients g/kg^†^		
Maize	560	650
Soybean	300	240
Til cake	40	—
Meat-bone meal	50	50
Oil	30	40
Shell grit	10.2	10.1
Di-calcium phosphate	6	6
Trace elements^#^	0.5	0.4
Salt	1.3	1.5
NSPase enzyme	0.5	0.5
Phytase 2500	0.2	0.2
DL-Methionine	1.3	1.3
L-lysine HCl	—	—
L-lysine sulfate	—	—

Chemical composition g/kg		
Crude protein^1^	222.3	191.6
Crude fiber	3.5	3.7
ME MJ/kg^2^	12.77	13.36
Total lysine^1&^	11.38	9.52
Total methionine^1^	4.87	4.49
Cysteine^1^	2.75	2.44
Arginine^1^	16.28	13.41
Threonine^1^	8.91	7.52
Tryptophan^1^	2.17	1.84
Calcium^1^	13.0	12.6
Total phosphorus^2^	7.2	6.2

^†^Additives added (mg/kg diet): vitamin AB_2_D_3_K 100, vitamin B complex 200, ethoxyquin 80, toxin binder 1000, liver stimulant 50, sodium bi carbonate 200, and bacitracin methylene di-salicylate 500.

^#^Contained (per kg) manganese 40 mg, iron, 30 mg, zinc 25 mg, copper 3.5 mg, iodine 0.3 mg, selenium 0.15 mg, and choline chloride 200 mg.

^1^Estimated in laboratory (amino acid analyses were performed by CJ CheilJedang Corporation, Gayang-dong, Gangseo-gu, Seoul, Korea); ^2^calculated values.

^&^The requirement of lysine of the experimental starter diet was met by replacing an equivalent amount of shell grit with 2 g/kg L-lysine.HCl and 3 g/kg L-lysine sulfate. In the finisher diet 1.2 g L-lysine.HCl and 1.9 g L-lysine sulfate replaced equivalent amounts of shell grit per kg basal diet to fulfill lysine requirement. The L-lysine.HCl contained 99% total lysine and 78% available lysine, and L-lysine sulfate contained 65% total and 51% available lysine.

**Table 2:** Chemical composition of L-lysine sulfate and L-lysine.HCl^†^.

Attribute	L-lysine sulfate	L-lysine.HCl
Active lysine content %	50.7	78.0–78.8

Metabolizable energy MJ/kg		
Swine	17.73	18.7
Poultry	17.3	16.7

Phosphorus %	0.16	Not detected

Additional amino acid %^#^		
Lysine	0.35	
Methionine	0.35	
Cysteine	0.10	
Threonine	0.28	
Tryptophan	0.14	
Arginine	0.38	
Isoleucine	0.33	

^†^Chemical analysis was performed by CJ CheilJedang Corporation, Gayang-dong, Gangseo-gu, Seoul, Korea.

^#^L-lysine.HCl did not contain any additional amino acid.

**Table 3:** Carcass traits and meat composition of broiler chickens fed diets supplemented with L-lysine.HCl and L-lysine sulfate.

Measurements		Source of supplemental lysine			SE	Significance (*P* value)
		Control^#^	L-lysine.HCl	L-lysine sulfate		
Live performance^1^						
Live weight g	Day 0	39	38	38	0.44	.127
	Day 40	1969^a^	2055^ab^	2156^b^	20.2	.006
Live weight gain g		1929^a^	2017^b^	2119^b^	20.1	.005
Food intake g		3752^b^	3712^b^	3612^a^	30.3	.028
Cumulative FCR		1.95^b^	1.84^b^	1.71^a^	0.002	.023
Hot carcass weight g		1516	1559	1557	29.4	.807
Eviscerated weight g		1217	1255	1261	22.5	.696
Carcass yield %		66.7	66.4	69.2	0.95	.431

Yield of carcass components g^2^						
Frame		251	285	286	6.28	.064
Breast		367^a^	427^b^	430^b^	8.0	.009
Legs		360	350	369	8.9	.671
Wings		114	115	105	2.4	.196
Giblets		106	112	115	3.6	.618

Yield of carcass components (%live weight)^2^						
Frame		18.9^a^	20.9^b^	20.7^b^	0.26	.015
Breast		27.9^a^	31.3^b^	31.3^b^	0.58	.05
Legs		27.1	25.6	25.8	0.36	.224
Wings		8.6^b^	8.5^b^	7.6^a^	0.14	.028

Meat composition g/100 g fresh weight (unless stated other wise)^2^						
Moisture		69.2	69.83	67.56	0.39	.61
Ash		5.1	4.9	4.5	0.35	.38
Fat		5.02^ab^	4.37^a^	5.97^b^	0.07	.024
Protein		20.68^a^	20.9^a^	21.97^b^	0.29	.005
Protein accretion g		405.9^a^	424.8^a^	494.3^b^	6.57	.0001

^1^Mean values of 8 replicates (*n* = 10 birds in each replicate).

^2^Mean values of 10 birds selected randomly from each treatment group.

Means bearing dissimilar superscripts varied significantly.

**Table 4:** Serum metabolites in broiler chickens fed diets supplemented with L-lysine.HCl and L-lysine sulfate.

Measurements^1^		Source of supplemental lysine			SE	Significance (*P* value)		
	Day	Control^#^	L-lysine.HCl	L-lysine sulfate		Diet	Day	Diet-day
Total protein g/L	0	44.7	44.5	44.8				
	40	36.1	38.2	34.8				
	Trial mean	40.4	41.4	39.8	0.89	.634	.0001	.623
Albumin g/L	0	28.3	28.9	28.7				
	40	18.3	18.8	14.5				
	Trial mean	23.3	23.9	23.6	1.01	.618	.0001	.623
Globulin g/L	0	14.4	15.5	16.2				
	40	17.8	19.4	18.9				
	Trial mean	16.1	17.5	17.5	0.47	.412	.0001	.868
Creatinine *μ*mol/L	0	60.8	54.8	57.3				
	40	62.2	62.5	58.1				
	Trial mean	61.5	58.3	57.7	3.73	.91	.634	.894
Uric acid *μ*mol/L	0	390.8	396.4	394.6				
	40	321.1	456.9	460.4				
	Trial mean	360.9	426.7	427.5	9.78	.11	.911	.001
Urea—N m mol/L	0	4.77	4.61	4.61				
	40	4.88	4.43	2.77				
	Trial mean	3.83	4.52	3.69	0.27	.91	.634	.894
Glucose m mol/L	0	9.14	8.55	8.85				
	40	8.17	8.32	8.82				
	Trial mean	8.66	8.44	8.68	0.21	.112	.241	.762
Cholesterol m mol/L	0	4.48	4.23	4.73			
	40	3.65	3.81	3.89			
	Trial mean	3.57	4.05	4.31	0.14	.112	.178	.348
Chloride m mol/L	0	104.3	106.4	105.2				
	40	101.3	100.9	98.8				
	Trial mean	102.9	103.7	102.1	0.32	.105	.0001	.088

^1^Mean value of 10 birds selected at random from the entire flock prior to the start of the experiment and then 10 birds selected randomly from each of the treatment group on day 40.

Means bearing dissimilar superscripts varied significantly.

**Table 5:** Intake (g/day unless stated otherwise) and metabolizability coefficients (g/g nutrient intake) of nutrients in broiler chickens fed diets supplemented with L-lysine.HCl and L-lysine sulfate.

Measurements^1^	Source of supplemental lysine			SE	Significance (*P* value)
	Control^#^	L-lysine.HCl	L-lysine sulfate		
Intake					
Food	93.8^b^	92.8^b^	90.3^a^	0.75	.028
Crude protein	19.5	19.4	19.2	0.11	.673
Metabolizable energy MJ^&^	1.22	1.21	1.18	0.011	.286
Phosphorus^&^	0.648^^a^^	0.64^a^	0.705^b^	0.007	.0001
Lysine^&^	0.988^b^	1.12^b^	1.44^c^	0.007	.0001
Methionine^&^	0.441^a^	0.442^a^	0.76^b^	0.003	.0001
Cysteine^&^	0.236^a^	0.234^a^	0.323^b^	0.009	.0001
Arginine^&^	1.39^a^	1.38^a^	1.72^b^	0.05	.0001
Threonine^&^	0.767^a^	0.759^a^	1.01^b^	0.007	.0001
Tryptophan^&^	0.188^a^	0.186^a^	0.313^b^	0.002	.0001
Metabolizability coefficient					
Dry matter	0.648^a^	0.684^a^	0.757^b^	0.015	.002
Organic matter	0.657^a^	0.713^a^	0.779^b^	0.009	.0001
Crude protein	0.516^a^	0.552^a^	0.661^b^	0.01	.003
N retention g/g intake	0.517^a^	0.551^a^	0.661^b^	0.015	.003
Protein accretion/g CP intake	0.527 ^a^	0.546 ^a^	0.603 ^b^	0.008	.001
Live weight gain/g CP intake	2.48^a^	2.59^a^	2.76^b^	0.026	.001
Live weight gain/MJ ME intake	39.4^a^	41.7^ab^	44.7^b^	0.41	.001
Live weight gain/g lysine intake	48.8^c^	45.1^b^	41.7^a^	0.44	.0001

^1^Based on data obtained during the metabolism trial conducted between day 32 to 38 (*n* = 10) selected at random from each treatment group.

Means bearing dissimilar superscripts varied significantly.

^&^ based on calculated values of dietary energy and total amino acid concentrations in the starter and the finisher diets.

**Table 6:** Estimation of fasting and endogenous nitrogen loss in the experimental broiler chickens.

Measurements^1^		Source of supplemental lysine			SE	Significance (*P* value)
		Control^#^	L-lysine.HCl	L-lysine sulfate		
Live weight g	Initial^2^	2016	2066	2086	30.4	.644
	Final^3^	1858^a^	1929^a^	2007^b^	30.2	.017
Change in live weight g		−159	−137	−80	3.6	.0001
Food intake g/day		67.2	70.4	67.1	2.68	.846
Fasting N excretion g/day		3.44	3.99	4.53	0.19	.109
Endogenous N excretion g/day		1.63	2.18	1.92	0.12	0.215
Serum protein g/L	Initial^#^	35.7	32.7	32.9	0.98	.314
	Final^&^	28.5	28.9	31.1	1.09	.001
Serum creatinine *μ*mL/L	Initial^#^	51.1	52.1	51.1	4.85	.709
	Final^&^	42.4	75.3	70.9	1.12	.0001

^1^Based on data obtained during the fasting trial conducted at tandem with the metabolism trial (*n* = 10) selected at random from each treatment group. The birds were fasted for 48 hours and then fed with tapioca starch for 6 days. The tapioca starch was supplemented with all the additives as it was with basal diet except the lysine.

^2^Indicates live weight after the fast ended and feeding of tapioca starch started.

^3^Indicates the end of feeding tapioca starch for 6 days.
